# Therapeutic control of leishmaniasis by inhibitors of the mammalian target of rapamycin

**DOI:** 10.1371/journal.pntd.0006701

**Published:** 2018-08-22

**Authors:** Fatemeh Khadir, Christopher R. Shaler, Ahmad Oryan, Patrick T. Rudak, Delfina M. Mazzuca, Tahereh Taheri, Jimmy D. Dikeakos, S. M. Mansour Haeryfar, Sima Rafati

**Affiliations:** 1 Department of Pathology, School of Veterinary Medicine, Shiraz University, Shiraz, Iran; 2 Department of Microbiology and Immunology, Western University, London, Ontario, Canada; 3 Department of Immunotherapy and *Leishmania* Vaccine Research, Pasteur Institute of Iran, Tehran, Iran; 4 Centre for Human Immunology, Western University, London, Ontario, Canada; 5 Lawson Health Research Institute, London, Ontario, Canada; 6 Division of Clinical Immunology and Allergy, Department of Medicine, Western University, London, Ontario, Canada; University of Notre Dame, UNITED STATES

## Abstract

Leishmaniasis is a serious global health problem affecting many people worldwide. While patients with leishmaniasis can be treated with several agents, drug toxicicty and the emergence of resistant strains render available treatments ineffective in the long run. Inhibitors of the mammalian target of rapamycin (mTOR) have been demonstrated to exert anti-pathogen properties. In this study, we tested the therapeutic efficacy of several mTOR inhibitors in controlling infection with *Leishmania major*. Rapamycin, GSK-2126458 and KU-0063794 were administered to BALB/c mice, which had received an intrafootpad injection of the parasite. Footpad swelling and parasite burden were assessed, and cytokine production by mouse splenocytes and phenotypic changes in draining lymph node cells were evaluated. Treatment with a clinically relevant dose of rapamycin or with GSK-2126458, but not with KU-0063794, dramatically lowered both the footpad swelling and the parasite load in the draining lymph node. Importantly, the employed dose of rapamycin did not kill the promastigotes *in vitro* as judged by 3-(4,5-dimethylthiazol-2-yl)-2,5-diphenyltetrazolium bromide (MTT) assays and electron microscopy. Moreover, the IL-4 production capacity of splenocytes harvested from infected mice that were treated with rapamycin was significantly reduced. Consequently, the IFN-γ:IL-4 production ratio was elevated, suggesting a T helper-type 1 (Th1)-skewed cytokine profile. Finally, the expression level of CD69, an early activation marker, on splenic and lymph node CD4^+^ and CD8^+^ T cells was enhanced in rapamycin-treated mice. Taken together, our findings suggest that select mTOR inhibitors may be used in therapeutic settings for the management of leishmaniasis. We propose that the beneficial effects of such inhibitors stem from their immunomodulatory properties. Therefore, the adjuvanticity of mTOR inhibitors may also be considered in vaccination strategies against *Leishmania* species.

## Author summary

The lack of effective vaccines and therapies for leishmaniasis along with the well-known resistance of the parasite to available agents prompted us to explore the effects of several mTOR inhibitors, including rapamycin, as potential therapeutics against *Leishmania (L*.*) major*, the classic *Leishmania* parasite strain. We found that systemic treatment with rapamycin and GSK-2126458, but not with KU-0063794, slows the progression of the disease and lowers the parasite burden in infected BALB/c mice. In addition, we observed more pronounced activation of CD4^+^ and CD8^+^ T cells in the draining lymph nodes in addition to a T helper 1 (Th1)-biased cytokine profile among the splenic cells of treated mice. Importantly, rapamycin blood levels achieved after *in vivo* treatment with this agent was far lower than the *in vitro* doses of rapamycin that killed the promastigote form of the parasite. Therefore, we propose that the impressive therapeutic efficacy of rapamycin, and perhaps GSK-2126458, against *L*. *major* are likely owed to the immunomodulatory properties of these agents. Based on our findings, mTOR may be an attractive target for the development of novel and potent anti-*Leishmania* agents.

## Introduction

Leishmaniasis is a significant public health concern with established clinical manifestations reported in more than 100 countries. The prevalence of leishmaniasis increases by about two million cases per year, and there are currently over 12 million individuals infected and more than 350 million people at risk [1,2]. The parasite is carried by the female phlebotomine sand fly and can infect a variety of mammalian species, including human [3]. Once in the infected host, *Leishmania* persists and multiplies within phagocytic cells such as macrophages. Clinically, *Leishmania* species. are responsible for three distinct forms of leishmaniasis, namely cutaneous, mucocutaneous and visceral leishmaniasis [4]. The disease severity and the clinical outcome depend largely on the species of *Leishmania* and the strength of the host response mounted against the parasite [5]. Individuals with underlying immunodeficiency, such as HIV/AIDS, are highly susceptible to disseminated forms of leishmaniasis and tend to have more severe manifestations [6,7]. Moreover, the genetic variation between *Leishmania* subspecies is one of the important factors in determining the disease outcome and is responsible for the diversity of the clinical manifestations encountered.

Historically, *Leishmania* species were classified into two groups, ‘old world’ and ‘new world’, based on their geographic distribution. *L*. *major*, *L*. *tropica* and *L*. *aethiopica* are considered ‘old world’, and *L*. *mexicana*, *L*. *amazonensis* and *L*. *guyanensis* are considered ‘new world’ strains [2,8]. While several common treatments are available for cutaneous leishmaniasis (CL), including pentavalent antimonial, meglumine antimoniate and sodium stibogluconate, many strains of *Leishmania* have developed resistance to these first-line treatments [9–11]. Further limiting the available treatments is the fact that several such drugs (*e*.*g*., pentavalent antimonial) are not appropriate for use in high-risk patients, such as the elderly, pregnant women and patients suffering from renal and cardiac diseases. In such cases, treatment requires long-term hospitalization, which in turn imposes a significant economic burden on healthcare systems in endemic regions [7,12]. Currently, amphotericin B is the most effective treatment for leishmaniais. However, it can be highly toxic, thus restricting its usage to only the most severe cases of leishmaniasis [13]. Amphotericin B is also expensive and therefore not available to the majority of patients with leishmaniasis in developing countries [14].

Although the mammalian target of rapamycin (mTOR) inhibitors, typified by rapamycin, are used in the clinic to prevent or delay graft rejection, recent work, including our own, demonstrated potent immune stimulatory properties for these inhibitors in the contexts of viral [15,16] and bacterial [17] infections and in anticancer immune surveillance [18]. Whether rapamycin and/or other mTOR inhibitors modulate anti-parasite immunity in general, and anti-*Leishmania* immunity in particular, is not clearly understood. In the current study, we investigated the efficacy of three different mTOR inhibitors, namely rapamycin, GSK-2126458 and KU-0063794, in a therapeutic setting during infection with *L*. *major*.

mTOR is a serine/threonine protein kinase that links environmental cues to a variety of pivotal biological functions and processes including but not limited to cellular growth, metabolism, survival, motility and autophagy [19,20]. mTOR is the catalytic subunit of two distinct multi-protein complexes known as mTOR complex 1 (mTORC1) and mTORC2. mTORC1 is composed of mTOR, regulatory-associated protein of mTOR (Raptor), mammalian lethal with Sec13 protein 8 (mLST8), proline-rich Akt substrate of 40 kilodaltons (PRAS40) and DEP domain-containing mTOR-interacting protein (Deptor). mTOR, mLST8 and Deptor are also present in mTORC2 complexes. However, mTORC2 additionally contains RPTOR independent companion of mTOR (Rictor), mammalian stress-activated protein kinase-interacting protein 1 (mSIN1) and protein observed with Rictor (Protor). Some of the signal inputs that regulate mTORC1 activity are relatively well known and belong to four general categories: nutrients, growth factors, energy levels and stress. Precise signals that are intercepted by mTORC2 are still a focus of intense research. mTORC1 controls transcription, ribosome biogenesis, mRNA translation and autophagy among other processes. mTORC2 controls cell survival and mediates the organization of the actin cytoskeleton [21].

Rapamycin potently inhibits mTOR after associating with its intracellular receptor, FK506-binding protein 12 (FKBP12) [22]. FKBP12-rapamycin complexes, once formed, bind to the FKBP12-rapamycin-binding (FRB) domain of mTOR and consequently inhibit its activity. Inhibition of mTORC1 appears to account for most of the known pharmacological effects of rapamycin. In fact, mTORC2 was originally identified as a rapamycin-insensitive entity since acute exposure to rapamycin failed to inhibit phosphorylation of Akt, a downstream target of mTORC2 [23]. However, subsequent studies demonstrated that long-term exposure to rapamycin could prevent the formation of new mTORC2 complexes [24]. It was more recently reported that rapamycin-induced longevity and insulin resistance are mediated by disruption of mTORC1 and mTORC2, respectively [25]. This indicates that both mTORC1 and mTORC2 can be targeted by rapamycin.

GSK-2126458 is a pyridyl sulfonamide inhibitor of both mTOR and phosphoinositide-3-kinase (PI3K), a key regulator of cellular growth [26]. KU-0063794 is a small molecule inhibitor of mTOR, which effectively blocks both mTORC1 and mTORC2, but not many other protein kinases including class-1 PI3Ks [27]. In this investigation, we have used the above inhibitors in parallel with rapamycin to explore the therapeutic potentials of mTOR inhibitors in the context of leishmaniasis. Our results demonstrate, for the first time to our knowledge, that inhibition of mTOR by rapamycin or GSK-2126458 decreases the parasite burden dramatically and reduce the morbidity of experimental leishmaniasis impressively. We also provide the evidence linking the observed benefits to the immune stimulatory properties of mTOR inhibitors. The biological and clinical relevance of our findings will be discussed.

## Materials and methods

### Chemicals, media and reagents

Rapamycin (*aka*. Sirolimus) was purchased from LC Laboratories (Woburn, Massachusetts), GSK-2126458 and KU-0063794 were obtained from Selleck Chemicals (Houston, Texas). Fetal Calf Serum (FCS) was obtained from Gibco (Montgomery County, Maryland). Gentamicin was obtained from Biosera (Heathfield, East Sussex). IL-4 and IFN-γ ELISA kits were purchased from eBioscience (San Diego, California). All other materials such as Schneider and RPMI-1640 media were purchased from Sigma-Aldrich (Oakville, Ontario).

### Ethics statement

All animal experiments were conducted following an animal use protocol (AUP# 2010–241) approved by Western University Animal Care and Veterinary Services (ACVS) in full compliance with the Canadian Council on Animal Care guidelines.

### Mice and parasite preparation

Adult 8 weeks female BALB/c mice were used in this study. Mice were purchased from Charles River Canada (St. Constant, Quebec), housed in a clean barrier facility at Western University. The *L*. *major* strain (Friedlin) was kindly provided by Dr. Jude Uzonna (University of Manitoba, Winnipeg, Manitoba, Canada). The infectivity of the parasites was maintained through repeated passages in BALB/c mice. The promastigotes were cultured at 26°C in Schneider medium (pH 7.4) supplemented with 10% heat-inactivated FCS and 50 μg/L gentamicin.

### Drug preparation

Ten mg of rapamycin was diluted in 1 mL DMSO and stored at -80°C. Prior to injections, the stock solution of rapamycin was diluted in a vehicle [phosphate-buffered saline (PBS) containing Tween 80 (5%) and Phosal 50 PG (95%)], and freshly prepared rapamycin was injected at indicated doses. Ten mg of GSK-2126458 and KU-0063794 were diluted in DMSO and stored at -80°C. The same vehicle was used as indicated above.

For *in vitro* experiments, rapamycin was diluted in PBS and used as a freshly prepared solution immediately.

### Determination of the half maximal inhibitory concentration (IC_50_) of rapamycin *in vitro*

The effect of various doses of rapamycin in the concentration range of 400 ng/mL to 5×10^4^ ng/mL (serial dilution at 1:2.5 ratio in PBS) was tested for *L*. *major* promastigotes in the logarithmic phase of the parasite growth (2×10^7^ parasites/mL) in sterile 96-well flat bottom culture plates (Orange Scientific, European Union). The parasites were incubated with the specified concentrations of rapamycin for 48 h. The drug’s potency was determined by MTT assay as we previously described [28]. Briefly, the MTT reagent (5 mg/mL) was added to each well, and plates were incubated for 4 h at 37°C. The plates were then centrifuged at 800 × *g* for 5 min followed by the removal of supernatant samples and addition of 100 μL DMSO to each well. The optical density (OD) of each well was measured at 540 nm and the concentration of the drug at which 50% inhibition in promastigote growth (ng/mL) was achieved (IC_50_).

### Determination of effective concentration of rapamycin (EC_50_) by parasite rescue assay

PMA-treated THP-1 human monocytic cells were seeded at 5×10^5^ cells per well in a 96-well culture plate (Orange Scientific) in RPMI-1640 complete medium supplemented with 10% heat-inactivated FCS. After 24 h incubation at 37°C in the presence of 5% CO_2_, the stationary-phase promastigotes of *L*. *major* were added to the cells at a parasite-to-cell ratio of 10:1. Free parasites were washed 24 h later with serum-free RPMI-1640 medium until no free parasite was visible under an inverted microscope. Infected THP-1 cells were treated with different concentrations of rapamycin ranging from 0.7 μg/mL up to 50 μg/mL (1:2.5 serial dilutions). The negative control consisted of THP-1 infected with the parasite without addition of any drug. After 48 h, cells that were infected and treated with the drug were washed with serum-free RPMI-1640 medium. RPMI-1640 with 0.05% Sodium dodecyl sulfate (SDS) was added to each well for cell lysis (20 μl/well). The plate was shaken for 30 seconds and complete Schneider culture medium (supplemented with 10% heat-inactivated FCS and 50 μg/L gentamicin) was added to each well (180 μl/well). The plates were incubated at 26°C for 72 h to transform the rescued live amastigotes into the promastigote form, and the potency of the drug to kill the intracellular *Leishmania* parasites was compared to that for untreated ones.EC_50_ (50% effective concentration) was determined using MTT test [29]

### Scanning electron microscopy (SEM) of the promastigotes

*L*. *major* promastigotes (2×10^7^ parasite/mL) were left untreated or treated with three different concentrations of rapamycin (5 μg/mL, 10 μg/mL and 20 μg/mL). For the primary fixation, the promastigotes were centrifuged at 1800 × *g* for 10 min. The supernatant was discarded, and the cells were suspended in a primary fixative, consisting of 2.5% EM-grade glutaraldehyde in PBS, for 2 h at room temperature on a rolling stage, followed by spreading the cells on coverslips. To prevent cells from drying out, the supernatant was removed, and cells were washed in 2 mL of H_2_O to completely remove the buffer salts. To dehydrate the samples, 30%, 50%, 70%, and 90% (v/v) ethanol in H_2_O was separately added to the coverslips, for 5 min, and washed three times with 100% ethanol. Coverslips were dried by a critical point dryer and mounted on SEM Silver stubs. The stubs were left overnight to dry. Samples were finally gold-coated (5–20 nm thickness) using a sputter coater and subsequently subjected to SEM microscopy [30–32]

### Transmission electron microscopy (TEM)

Uninfected and infected THP-1 cells that were left untreated or were exposed to rapamycin using the EC_50_ concentration of the drug were ultra-structurally visualized after TEM electron microscopy fixation method. Promastigotes were also left untreated or treated with the IC_50_ concentration of rapamycin. The cells and promastigotes were then spun at 1800 x *g* for 10 min. The supernatant was removed, and the cells were fixed for 1 h at 4°C in 2.5% glutaraldehyde in 0.1 M cacodylate buffer, pH 7.2, as the primary fixative. The cells were rinsed twice in 0.1 M cacodylate buffer and post-fixed in 1% osmium tetroxide for 1 h. To improve the rate of penetration, samples were agitated slowly on an electric turn-table and subsequently dehydrated in a graded series of acetone. To improve Epon penetration, the samples were left in an equal volume of propylene oxide and Epon resin overnight and then in fresh 100% Epon for another 24 h. The blocks were finally polymerized in fresh Epon resin.

One μm-thick sections were cut with a glass knife using a Reichert ultramicrotome OmU3, stained with methylene blue, and observed under a light microscope to determine the perfect place of the section. Pale gold sections (80-nm-thick) were then cut and stretched with chloroform vapor to relieve compressional artifacts. Sections were routinely picked up on 200-mesh copper grids, having carbon stabilized formvar support films and stained by immersion in saturated uranyl acetate for 10 minutes. Subsequently, they were washed in several changes of running distilled water, immersed in lead citrate stain for 5 min, and finally washed extensively with running distilled water and air-dried. The sections were viewed and photographed using a Philips EM-10C transmission electron microscope at conventional voltages (60 to 100 Kv) [33].

### Infection of mice and treatment schedule

*L*. *major* parasites were cultured at 26°C in Schneider medium supplemented with 10% FCS. The metacyclic promastigotes were obtained using Ficoll 400 gradient centrifugation from stationary-phase parasites [34]. Mice were randomly assigned to 8 groups (at least n = 3 per group), including two control cohorts receiving vehicle in the amounts corresponding to high-dose and low-dose treatments. Mice were injected in the footpad with 2 × 10^6^ metacyclic promastigotes/50 μL. Treatment was started at three weeks post-infection with high doses (10.2 μg/dose) or low doses (1.5 μg/dose) of rapamycin, GSK-2126458 or KU-0063794 [35] as indicated. Daily treatments were given intraperitoneally (*i*.*p*.) for 10 days. Control groups received vehicle *i*.*p*. during 10 days of treatment; mice were sacrificed for isolation of lymph nodes and spleens. Blood specimens were also collected through terminal cardiac puncture, in which rapamycin levels were quantified using a commercially available kit.

### Footpad thickness measurement

Changes in infected footpad thickness were measured by a digital caliper (World Precision Instruments, Sarasota, Florida), starting on the day 1 of parasite inoculation until day 31 post-infection. The thickness of the non-infected footpad was also recorded and used as the background. Footpad pictures in all groups were also taken at the end of each experiment.

### Quantification of parasite burden in popliteal lymph nodes

The absolute copy number and relative burden of parasites in the popliteal lymph nodes (pLNs) were measured by quantitative PCR (qPCR). Genomic DNA (gDNA) of whole lymph nodes were extracted using the DNeasy Blood & Tissue kit from Qiagen Sciences (Germantown, Maryland) according to the manufacturer’s instructions. The concentration and purity of extracted DNA were assessed using a Nanodrop (ND-1000) spectrophotometer. For each PCR reaction, 30 nanograms of total gDNA and 500 nM of primers targeting the kinetoplastid minicircle regions RV1 and RV2 (Forward: 5' CTTTTCTGGTCCCGCGGGTAGG3'; Reverse: 5' CCACCTGGCCTATTTTACACCA3'; Sigma-Aldrich) were used. Serial dilutions of *L*. *major* gDNA in the range of 10^2^−10^7^ parasites were used to generate a standard curve. Ten μL of 2X power SYBR green PCR master mix (Applied Biosystems) was added for a final volume of 20 μL per reaction and the number of cycles (40) was set according to the manufacturer’s instructions. PCR amplifications and melt curve analyses were conducted using the StepOne Plus Real-Time PCR instrument and software (Applied Biosystems). Resulting cycle threshold (Ct) values were normalized to the mammalian housekeeping gene glyceraldehyde 3-phosphate dehydrogenase (GAPDH) (500 nM Forward: 5’CGTCCCGTAGACAAAATGGT3’; 500 nM Reverse: 5’TTGATGGCAACAATCTTCAC3’; Sigma-Aldrich) and delta-delta Ct (ΔΔCT) values were determined using the following calculations: ΔCt _sample_ = Ct _target_−Ct _reference (GAPDH)_ and ΔΔCT = ΔCT(_sample_)−ΔCT(_vehicle_). Fold change differences compared to the vehicle-treated groups were calculated using the formula: Fold change (normalized to vehicle) = 2^-(ΔΔCT)^ [36]. All samples were run in technical duplicates.

### Preparation of parasite lysate

A total of 10^8^ stationary-phase parasites per mL were subjected to repeat freezing in liquid nitrogen and thawing in a 37°C water bath until the parasites were lysed completely. The protein content of the parasite lysate was quantitated using a Pierce BCA Protein kit (Thermo Fisher Scientific, Waltham, Massachusetts).

### Measurement of cytokines by ELISA

Control and treated mice were sacrificed at the end, namely on day 31 post-infection. Splenic single cell suspensions were prepared followed by treatment with ACK (Ammonium-Chloride-Potassium) lysis buffer to remove erythrocytes. Cells were washed, resuspended in RPMI-1640 medium (Gibco, Montgomery County, Maryland) containing 10% FCS, 10 mM HEPES and 50 μg/mL of penicillin, and seeded at 1 × 10^5^ cells/250 μL medium in a U-bottom polystyrene microplate. Cells were stimulated with 10 μg/mL of *Leishmania* lysate [37]. As positive control, cells were stimulated with a 1:20 dilution of the 145-2C11 hybridoma culture supernatant, which approximates 0.5 μg/mL of an agonistic anti-mouse CD3 mAb. Culture supernatants were harvested 24 and 72 h later, and their IFN-γ and IL-4 contents were determined by ELISA kits purchased from eBioscience (San Diego, California).

### Cytofluorimetric evaluations

A 1-mL Dounce tissue grinder was used to prepare mononuclear cell (MNC) suspensions from sentinel and control popliteal lymph nodes draining infected and uninfected footpads, respectively. Splenic MNCs were prepared using a glass tissue homogenizer.

Freshly isolated MNCs were subjected to cytofluorimetric evaluation. A total of 1 × 10^6^ cells was seeded in a U bottom plate, and nonspecific Fc receptor-mediated binding of antibodies was blocked by exposing the cells on ice to 20 μL undiluted culture supernatant of the 2.4G2 hybridoma producing anti-mouse CD16/32 mAb. Following Fc blockade, cells were washed and stained with fluorochrome-conjugated mAbs to several cell surface molecules, including anti-CD3ε-PE Cyanine 7 [clone 145-12C11 (eBioscience)], anti-CD8α-APC [clone 53–6.7 (eBioscience)], anti-CD4–FITC [clone GK1.5 (eBioscience)] and anti-CD69-PE [clone H1.2F3 (BD Pharmingen)]. All antibodies were diluted in 2% FCS in PBS, and cells were stained for 30 min on ice, washed thoroughly, and evaluated using a BD FACSCanto II flow cytometer (BD Biosciences, Mississauga, Ontario). Following acquisition, the expression of the above surface markers was analyzed using FlowJo software (Tree Star, Ashland, Oregon).

### Statistical analysis

GraphPad Prism (version 6.0; Graph Pad Software, Inc. 2007, San Diego, California) was utilized for statistical analyses. Student’s *t*-test and ANOVA were performed, to determine whether the observed differences were statistically significant. The *P* values of less than 0.05 were considered significant.

## Results

### Treatment of *Leishmania*-infected mice with rapamycin or GSK-2126458 diminishes footpad swelling and lesions

**Fig 1:**
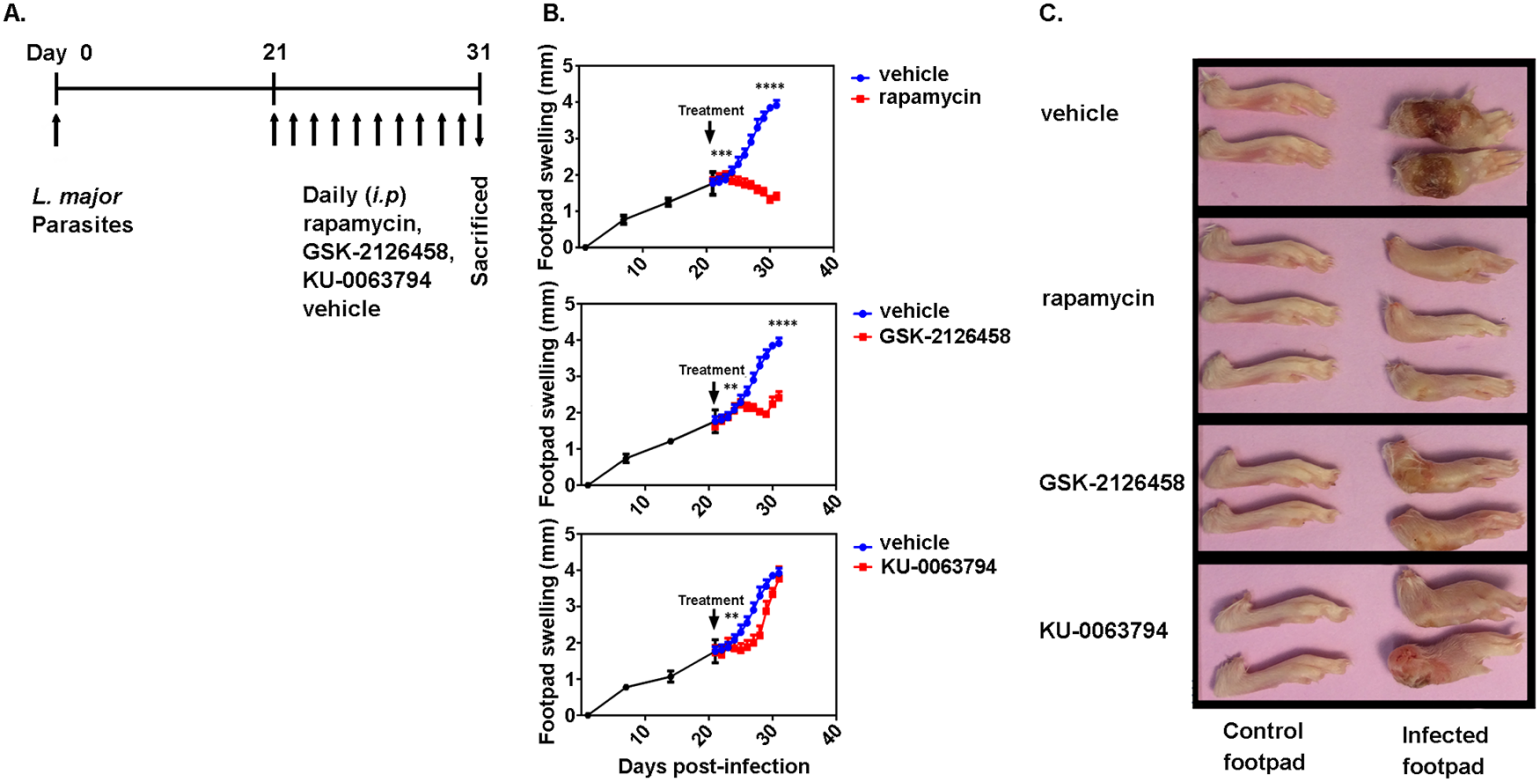
The effect of treatment with mTOR inhibitors on footpad swelling of *L*. *major*-infected mice. BALB/c mice were inoculated in their footpad with *L*. *major*. After 21 days, mice were treated *i*.*p*. with vehicle (n = 9), rapamycin (10.2 μg/dose/day, n = 9), GSK-2126458 (10.2 μg/dose/day, n = 9), or KU-0063794 (10.2 μg/dose/day, n = 9). The timeline for infection and treatment with indicated mTOR inhibitors is schematically depicted **(A)**. Control and infected footpads are shown at the experimental end point (day 31 post-infection) before (shown as black) and after treatment with mTOR inhibitors (shown as red) or vehicle (shown as blue) **(B)**. The observed changes in footpad swelling and lesions over the course of the disease/treatment are graphed form one representative experiment **(C)**. Data are pooled from 3 experiments, each of which typically consisted of at least 3 mice/group. Error bars represent standard error of the mean (SEM). The difference in footpad swelling was analyzed using multiple comparisons, two-way ANOVA at each time point. **: *p*< 0.01***: *P*<0.001 **** *P*<0.0001.

Mice were infected with *L*. *major* and then treated daily with either 1.5 μg or 10.2 μg/dose/day of rapamycin, GSK-2126458, KU-0063794, or with vehicle according to the regimen that is schematically depicted in Fig 1A. Footpad thickness was monitored starting on day 1 post-infection. Slight swelling of the infected footpads was evident as early as on day 7 post-infection and intensified gradually afterwards. Treatment with high-dose rapamycin, which was started on day 21, led to a significant decline in footpad thickness started from day 26 (*p*<0.001), rapamycin-treated mice showed significant changes were compared with vehicle-treated controls at the end point (day 31) (*p*<0.0001) (Fig 1B). Similarly, there was a significant decrease in footpad swelling in mice treated with high-dose GSK-2126458 starting from day 27 (*p*<0.01), the treated group showed significant decrease in footpad swelling at the end point as well (*p*<0.001). There was a significant decrease in footpad swelling at the 27 days post infection in KU-0063794 treated mice (*p*<0.01). No significant changes have been detected in KU-0063794 treated group in comparison of the vehicle control at the end point of 31 days as illustrated in Fig 1B. Also, interestingly, animals that were treated with 10.2 μg daily doses of rapamycin or GSK-2126458 had milder lesions (Fig 1C). In contrast, footpad swelling, and lesion formation was not affected by treatment with 1.5 μg/dose of rapamycin, GSK-2126458 or KU-0063794 (S1A and S1B Fig). Collectively, the above findings demonstrate that treatment with select mTOR inhibitors slows the progression of cutaneous Leishmaniasis.

### Rapamycin and GSK-2126458 lower the parasite burden in the lymph nodes of *L*. *major*-infected mice

**Fig 2:**
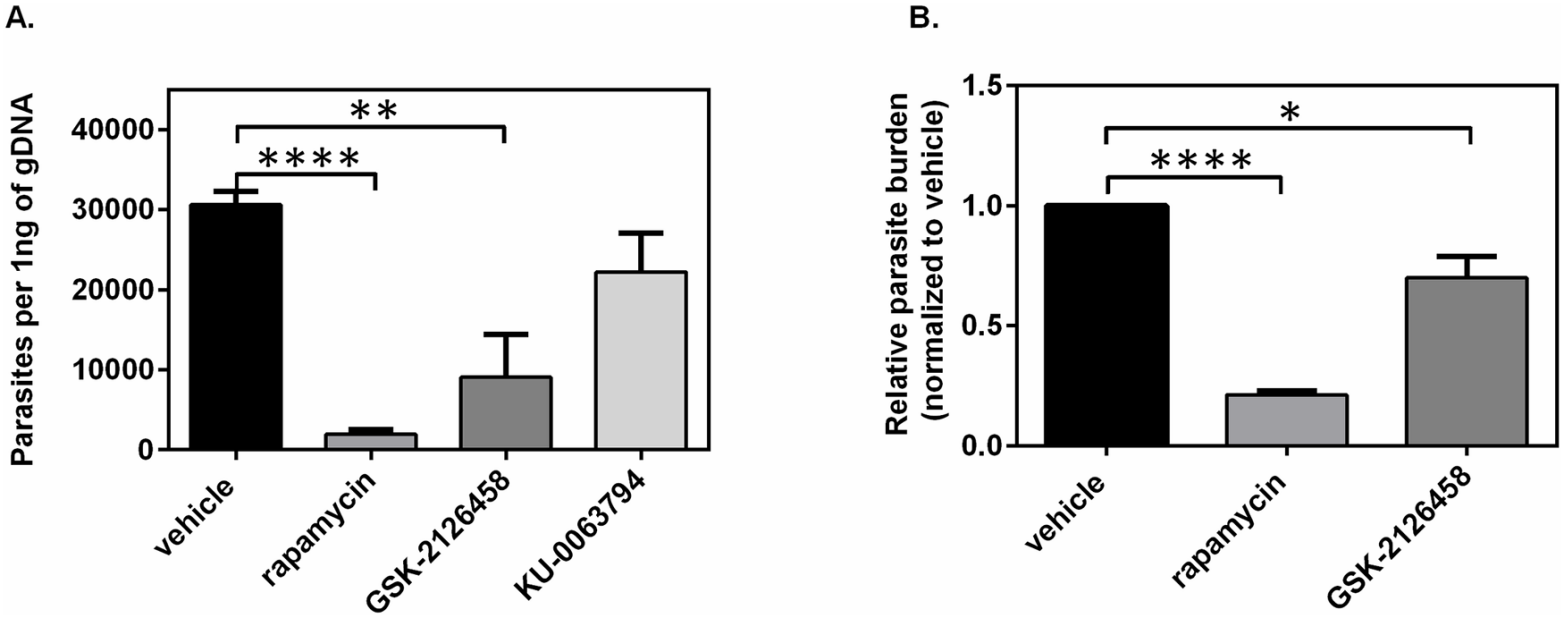
Enumeration of *Leishmania* parasite burden in BALB/c mice treated with vehicle or with daily high doses of mTOR inhibitors. Following the treatment schedule highlighted in Fig 1A, parasite burden in the draining popliteal lymph nodes (pLN) of the infected footpad was determined by qPCR of bulk genomic DNA. Absolute copy numbers of *Leishmania* RV1-RV2 in the pLN of mice treated with vehicle or 10.2 μg/dose of rapamycin, GSK-2126458 or KU-0063794 were calculated via a standard curve and values are presented as number of parasites per ng of gDNA **(A)**. In parallel, gDNA for the mammalian GAPDH housekeeping gene was quantified and normalized parasite numbers in mTOR inhibitor-treated mice were quantified relative to that of vehicle-treated mice using the ΔΔCt method **(B)**. The error bars represent the standard error of the mean (SEM), data were pooled from three different experiments, n = 9. Statistical differences in parasite burden between groups were determined using one-way ANOVA, Student *t*-test was done between untreated group (vehicle) and treated group. *: *p*< 0.05; **: *p*< 0.01; ****: *p*< 0.0001.

Upon the completion of our therapeutic regimen in *L*. *major*-infected mice, remaining parasite burden was assessed by qPCR of total genomic DNA from popliteal lymph nodes using primers for the RV1-RV2 kinetoplastid minicircle region. Total copy number of *Leishmania* parasites per nanogram of genomic DNA were calculated based on a standard curve and are presented in Fig 2A and S2 Fig. Parasite copy number following treatment with 10.2 μg/dose of rapamycin was significantly reduced compared to treatment with vehicle (*p*<0.0001) and demonstrate that parasite propagation to the draining lymph nodes of the infected footpad is strongly prevented by rapamycin (Fig 2A). Likewise, the parasite load in the lymph nodes of mice treated with GSK-2126458 were significantly diminished in comparison with mice treated with vehicle (*p*<0.01). However, we observed no statistical differences in the total number of parasite copies in lymph nodes between vehicle-treated and KU-0063794-treated subjects. Of note, mice treated with low doses (1.5 μg/dose) of any of the tested mTOR inhibitors displayed no significant changes in resulting parasite load in the lymph nodes (S2 Fig).

Relative parasite burden between groups was determined by normalizing parasite copy number to the Ct values of the mammalian GAPDH housekeeping gene and calculating fold change differences using the ΔΔCT method. Consistent with our findings using absolute copy number, the relative parasite burden in the popliteal lymph nodes of mice treated with 10.2 μg/dose of rapamycin or GSK-2126458 were significantly lower than that of mice treated with vehicle (*p*< 0.0001 and *p*< 0.05, respectively; Fig 2B). Taken together, we conclude that therapeutic administration of select mTOR inhibitors, including rapamycin and GSK-2126458, can dose-dependently control *L*. *major* dissemination *in vivo*.

### The *in vitro* parasiticidal effect of rapamycin on *L*. *major* does not appear to account for its *in vivo* efficacy

**Fig 3:**
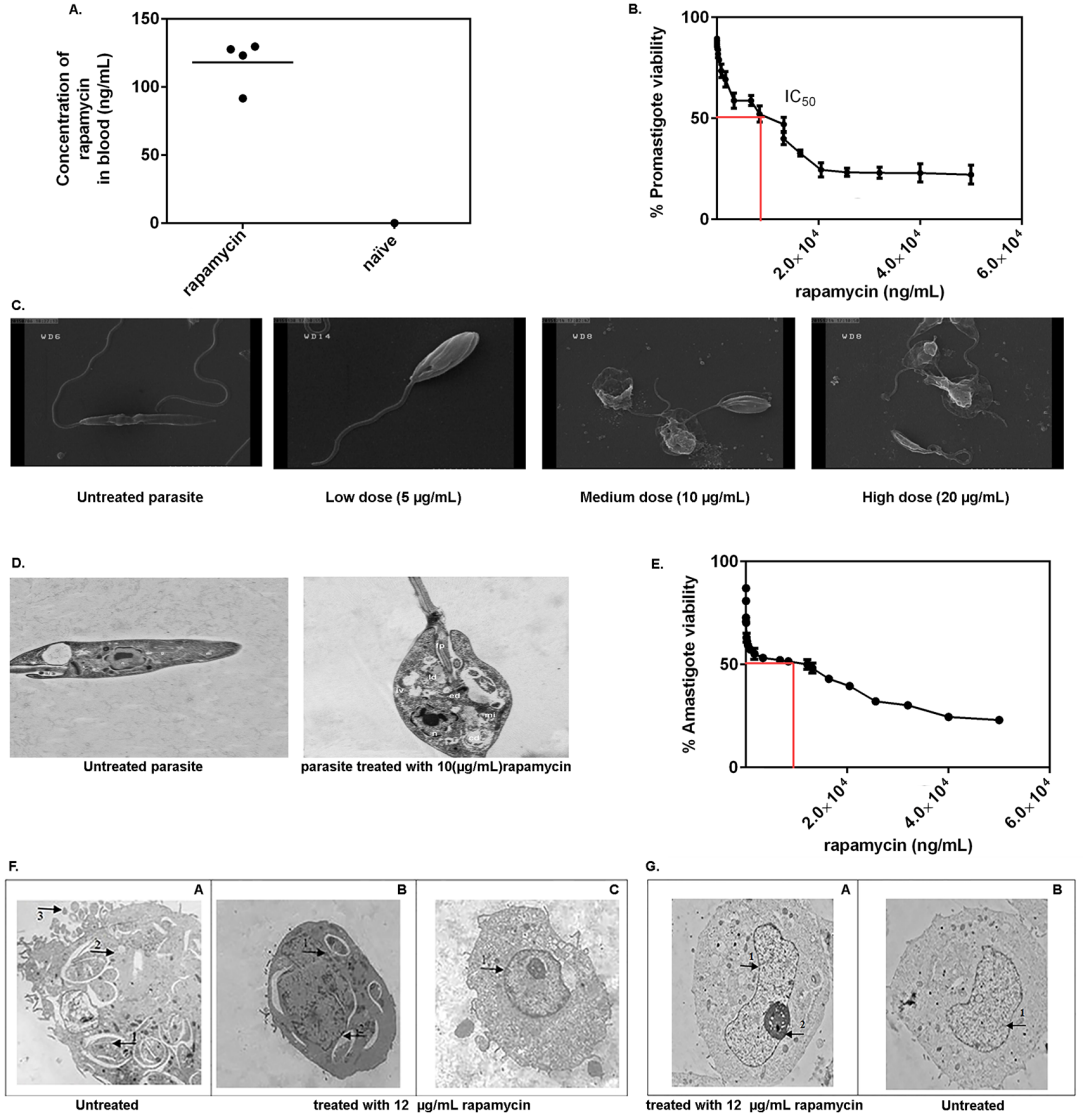
*In vivo* concentrations of rapamycin do not exhibit direct toxic effect on *Leishmania* promastigotes *in vitro*. *In vivo* serum concentration of rapamycin was determined after 10 days of daily *i*.*p*. treatment with 10.2 μg/dose (n = 4) **(A)**. Twenty million stationary phase parasites/mL were treated with different doses of rapamycin (0–50 μg/mL) and IC_50_ was determined by MTT assay (**B**). Based on determined IC_50_, stationary phase parasites treated for 48 h with low, medium and high doses of rapamycin (5, 10 and 20 μg/mL), and then subjected to SEM electron microscopy, the scale was 7μm. (**C**). Parasites treated with 10 μg/mL of rapamycin were subjected to TEM electron microscopy (**D**) The scale was 500 nm. n: nucleus; fp: flagella pocket; mi: mitochondrion; g: golgi apparatus; k: kinetoplast; iv: intracellular vacuole; c: carbohydrate droplet; ld: lipid droplet; ed: electron dense structure. **Fig 3E**: Effect of Rapamycin on the THP-1 cells infected with stationary-phase *L*. *major* promastigotes. The growth of the surviving parasites was determined by parasite rescue assay. EC_50_ of rapamycin for infected THP-1 was 12 μg/mL. **Fig 3F**: A) Transmission ultra-micrograph of *L*. *major* amastigotes in the cytoplasm within THP-1 cells. Numerous intracellular parasites were detected in a single THP-1 cell. Pseudopodia of the cell are visible on the surface.1: intracellular parasite, 2: intracellular vacuole, 3: pseudopodia formation (shown as arrows) B) A THP-1 cell infected with *L*. *major*, treated with the EC_50_ dose of rapamycin (12 μg/mL). The cells were infected with the parasite and treated with rapamycin and incubated for 24 h as previously described. Three injured amastigotes within the infected cell. 1–2: intracellular parasite (shown as arrows) C) THP-1 cell infected with *L*. *major* and treated with rapamycin. The cells were treated with EC_50_ dose of rapamycin and incubated for 48 h and fixed for TEM. No trace of *Leishmania* amastigote, pseudopodia formation on the surface, or vacuole formation was observed. The intracytoplasmic organelles of the cell remained intact. 1: nucleus (shown as arrow) The scale was 1μm. **Fig 3G**: Ultra micrograph of THP-1 cell after treatment with 12 μg/mL of rapamycin with no signs of toxicity and normal THP-1 cells. A) THP-1 cells treated with 12 μg/mL of rapamycin 1: nucleus 2: nucleolus (shown as arrows) B) Normal THP-1 cell 1: nucleus (shown as arrow) All The experiment was repeated two times. The scale was 1μm.

We quantitated the blood concentration of rapamycin in 4 mice that had received high-dose rapamycin and in one untreated naïve mouse as a control (Fig 3A). In addition, the impact of *in vitro* treatment with a wide range of rapamycin doses on *L*. *major* promastigotes and amastigotes was evaluated (Fig 3B and 3E). Our *in vitro* studies showed that rapamycin is able to kill the promastigotes and amastigotes only at high doses. It is worth to mention that this amount is far higher than employed doses for *in vivo* study found in the circulation of treated mice (Fig 3A, 3B and 3E). The IC_50_ of rapamycin for *L*. *major* promastigotes was 10 μg/mL and the EC_50_ for *L*. *major* amastigotes was 12 μg/mL while the mean blood concentration of rapamycin in treated groups was 117.875 ng/mL. To further examine the direct *in vitro* effect of rapamycin on the promastigotes, three different doses of rapamycin were selected based on our IC_50_ results and tested for their impact on the morphology of the parasites by electron microscopy. These consisted of low (5 μg/mL), intermediate (10 μg/mL) and high (20 μg/mL) doses of the agent (Fig 3C). We also included *L*. *major* without any treatment in these experiments as a control. Distinctive ultrastructural alterations, together with changes in shape and size of the cells, were observed upon rapamycin treatment in a dose-dependent manner. The parasites were shrunk in size in the treated groups in comparison with the control group. The body of the parasites was also cracked up, and the cells became round. Exposure to 20 μg/mL of rapamycin *in vitro* resulted in drastic changes in shape and size of *L*. *major* and the cells were damaged as evidenced by the presence of cellular debris around the parasites’ body. The extent of size reduction and roundness of the cells upon exposure to 10 μg/mL and 5 μg/mL of rapamycin also pointed to a dose-dependent effect.

The ultrastructural properties of untreated *L*. *major* promastigotes and those exposed to the IC_50_ dose are shown in Fig 3D. The noted decrease in the cell size and the internal organelles together with formation of vacuoles in the cytoplasm confirmed that rapamycin had a direct negative effect on the promastigotes (Fig 3D). The effect of different doses of Rapamycin on PMA-treated THP-1 cells infected with *Leishmania* parasite for 48 h are shown in Fig 3E. EC_50_ (12 μg/mL) of rapamycin for the amastigote forms of *L*. *major* was determined by parasite rescue assay. The ultrastructural observation of *L*. *major* amastigote forms treated with EC_50_ doses of rapamycin, and control groups (infected THP-1 cells with no treatment) are shown in Fig 3F. For better observation of drug efficacy, the treated cells were collected once at 24 h (Fig 3F-B) and again at 48 h (Fig 3F-C). The TEM results demonstrated that the frequency of injured amastigotes within THP-1 cells treated with rapamycin in 24 h was higher in comparison with the control group as shown in Fig 3F-A (infected cells without any treatment). Infected cells, which were treated with rapamycin showed no visible parasites after 48 h. The absence of amastigotes within THP-1 cells once infected and then treated with rapamycin suggests that the cells no longer act as a host for the *Leishmania* parasites. A THP-1 cell exposed to the EC_50_ dose of the drug and after 48 h of incubation is shown in Fig 3G. The treated showed no sign of toxicity as demonstrated in Fig 3G-A.

Our *in vitro* observations strongly suggest that while exposure to rapamycin at certain doses can inflict morphological damage upon the intracellular and extracellular parasite, similar doses were not achieved following *in vivo* rapamycin treatment in our mouse model. In fact, the dose equivalent to the mean concentration of rapamycin in the blood of treated mice did not alter either the morphology or the viability of the parasite. The adverse changes observed in the aftermath of rapamycin treatment *in vitro* emerged with microgram concentrations of the drug, which is much higher than the effective concentration *in vivo* (Fig 3A, 3B and 3E).

### Splenocytes of rapamycin- and GSK-2126458-treated mice exhibit a Th1-skewed response to *Leishmania* lysate

**Fig 4:**
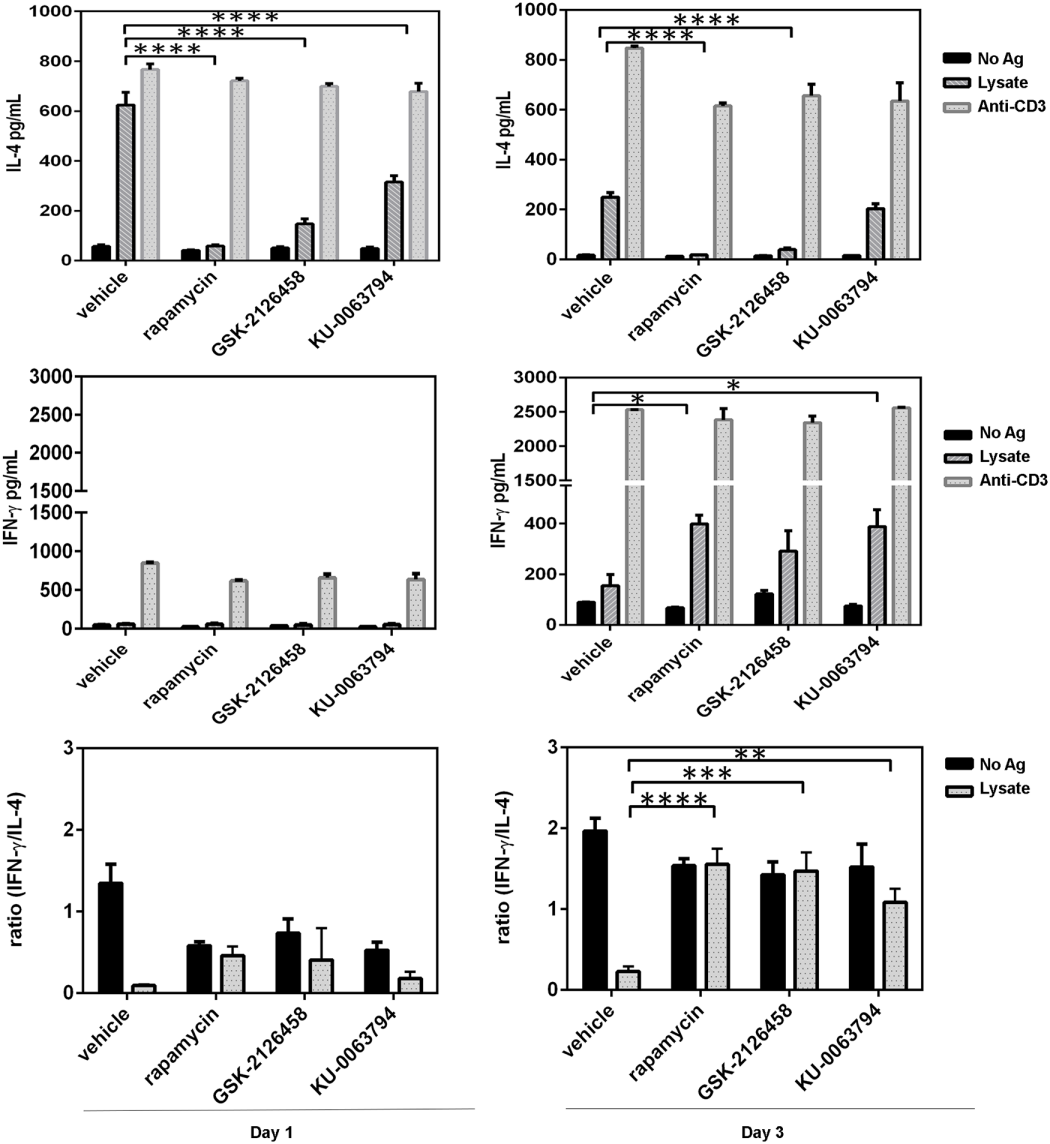
Splenocytes from *Leishmania*-infected and mTOR inhibitor-treated mice demonstrate Th1-polarized cytokine bias. Splenocytes from *Leishmania*-infected mice were left untreated or exposed to 10 μg/mL lysate preparation or a 1:20 dilution of culture supernatant obtained from an anti-CD3 mAb-producing hybridoma. Culture supernatants were collected 24 and 72 h after stimulation with lysate or anti-CD3 mAb for the measurement of IL-4 (**A**) and IFN-γ (**B**). The IFN-γ/IL-4 ratio was also determined at indicated time points (**C**). Error bars represent standard error of the mean (SEM) and the data were collected from three different experiments (n = 9). Statistical differences in cytokine production between groups were evaluated using one-way ANOVA, comparison of vehicle control and the other treatments groups at the end of time point were determined, using a Student’s *t*-test. *: *p*<0.05; **: *p*<0.001; ****: *p*<0.0001.

Next, we explored a potential bias towards Th1- or Th2-type cytokine responses following treatment with rapamycin or GSK-2126458. We focused on IFN-γ and IL-4, which typically represent Th1- and Th2-type cytokines, respectively. Splenocytes isolated from mTOR inhibitor and vehicle-treated mice were stimulated *ex vivo* with a crude parasite lysate preparation, and supernatants were collected after 24 h (Fig 4A, 4B and 4C and S4A, S4B, S4C Fig, left panel) and 72 h (Fig 4A, 4B and 4C and S4A, S4B, S4C Fig, right panel). An agonistic anti-mouse CD3 mAb was used as a positive control. The IL-4 content of culture supernatants was significantly lower in the rapamycin treatment groups than in the control groups (*p*<0.0001). The GSK-2126458 treatment groups also showed significantly lower IL-4 levels at both 24 h and 72 h time points (*p*< 0.0001, Fig 4A). There were also significant IL-4 changes upon treatment with KU-0063794 high dose at only 24 h time point (*p*<0.0001) (Fig 4A). High levels of IL-4 were detectable in cultures containing splenocytes prepared from vehicle- and low-dose mTOR inhibitor-treated mice (S3A Fig). Moreover, IFN-γ production did not significantly change at the 24 h time point (Fig 4B). However, high-dose rapamycin treatment was associated with increased IFN-γ production at the 72 h time point compared with vehicle control (*p*< 0.05) (Fig 4D, right panel). Surprisingly, KU-0063794 was significantly increase IFN-γ production at 72 h time point (*p*< 0.05, right panel)

When we determined the IFN-γ/IL-4 ratio at 24 h post-stimulation, no differences were noticeable, likely due to very low concentrations of IFN-γ at this specific time point. However, there was a significant increase in IFN-γ/IL-4 ratio in the rapamycin, GSK-2126458 and KU-0063794 treatment groups at 72 h time point (*p*<0.0001, *p*<0.001 and *p*<0.01) (Fig 4C right panel). Collectively, these findings suggest Th1-skewed responses in rapamycin, GSK-2126458 and KU-0063794 -treated mice in compare to vehicle, which may contribute to the therapeutic efficacy of these inhibitors *in vivo*.

### The effect of mTOR inhibitors on T cell activation *Leishmania* -infected mice

**Fig 5:**
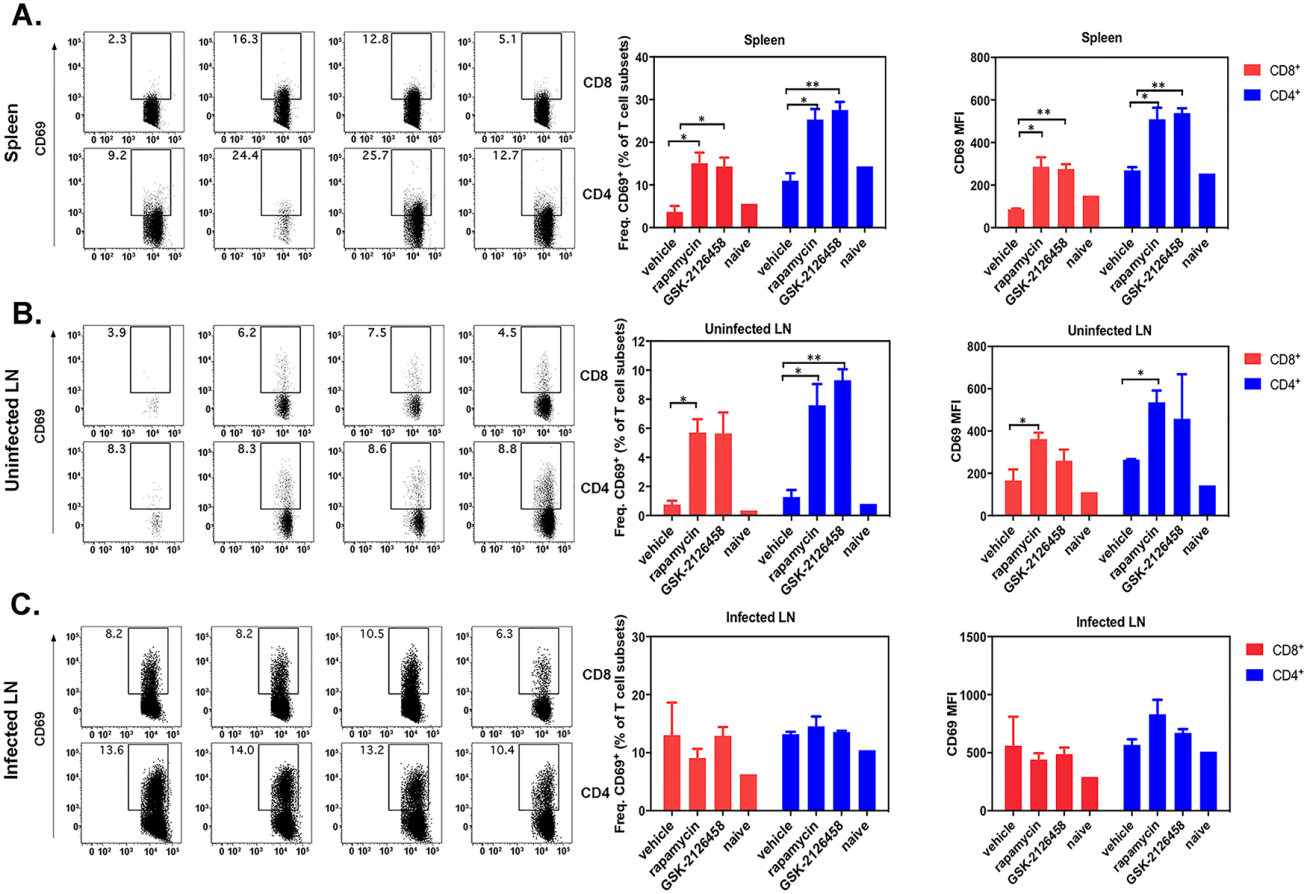
Treatment with mTOR inhibitors does not impair T cell activation in the lymph nodes and spleen of *Leishmania*-infected mice. Following 10 days of daily *i*.*p*. treatment with mTOR inhibitors or vehicle, the activation status of CD4^+^ and CD8^+^ T cells was determined. Representative FACS plots (left) and pooled data (right) depict the frequency of CD69^+^ cells as well as the MFI of CD69 expression among TCRβ^+^CD4^+^ or TCRβ^+^CD8^+^ cells in the spleen (**A**), in the popliteal lymph nodes of the uninfected footpad (**B**) and the draining popliteal lymph nodes of the infected footpad (**C**). Error bars represent SEM from two experiments (n = 8). Statistical differences in were determined by Student’s *t*-test *: *p*<0.05; **: *p*<0.001; ****: *p*<0.0001.

We next investigated the activation status of CD4^+^ and CD8^+^ T cells in two key immune compartments. Ten days after mTOR inhibitor or vehicle treatment, spleens and popliteal lymph nodes were extracted from *Leishmania*-infected mice and subjected to cytofluorimetric analysis. We examined the expression of CD69, an activation marker, on CD4^+^ and CD8^+^ T cells in the spleen and popliteal lymph nodes. Specifically, in the spleen, the frequency, and the mean fluorescence intensity (MFI) of CD69 were increased on both CD4^+^ and CD8^+^ T cell subsets in mice that had been treated with rapamycin or GSK-2126458 when they were compared with vehicle-treated *Leishmania*-infected or naïve mice (Fig 5A). Similar results were obtained for splenic and popliteal lymph node CD4^+^ and CD8^+^ T cells in uninfected footpads (Fig 5B). In contrast, CD69 expression did not increase on either T cells subset when the popliteal lymph nodes draining the infected footpad were examined (Fig 5C). Importantly, while these responses did not increase in the vehicle control, they did not significantly decrease either, suggesting that mTOR inhibition did not impair T cell activation in response to *Leishmania*. Taken together, these results suggest that treatment with mTOR inhibitors, at doses employed in this study, does not impair T cell-mediated anti-*Leishmania* immunity, and may instead promote T cell activation in distal lymphoid organs.

## Discussion

Although rapamycin was initially considered an antifungal agent, it is now mainly used as an immunosuppressive agent in allograft recipients [38]. The relatively recent findings that rapamycin and its analogs may boost immune responses to viral pathogens such as lymphocytic choriomeningitis virus (LCMV)[15] and vaccinia virus [15,16] have stirred enthusiasm in the scientific community for their potential application in a broader range of infections. Notwithstanding, a precious little is known about whether or how mTOR inhibitors may affect anti-parasite immunity.

TOR is known to play a role in autophagy, in survival within phagosomes and in growth of *Leishmania* parasite [39]. TOR1 and TOR2 are two virtual genes in *L*. *major* that are involved in stress response [40]. Given its important roles in virulence of *Leishmania* parasites [41], TOR1 may be considered a potential drug target [42]. We hypothesized that rapamycin and other agents that inhibit mTOR, alone or together with PI3K, may inhibit the parasite propagation and/or modulate anti-parasite immune responses to stop the progression of the disease. It is worth mentioning that, GSK-2126458 was chosen because of its dual PI3K/mTOR inhibitory activity. KU-0063794 was chosen due to its inhibitory effect on both mTORC1 and mTORC2. Certain anti-leishmanial effects for mTOR inhibitors have been reported [42,43]. An interesting *in vitro* study revealed the effect of rapamycin on IL-12/IL-10 axis in a *L*. *donovani* infection model [43]. However, to our knowledge, rapamycin, its analogs and dual mTOR/PI3K inhibitors have not been used in *in vivo* models of infection with *Leishmania* spp.

It has been recently shown that rapamycin can act as an allosteric inhibitor of the mTORC1, which binds to the intracellular protein FKBP12 and induces autophagy in nanomolar doses [44]. According to a study conducted by Martin *et al*., high-dose rapamycin can induce apoptosis due to caspase activation, and both micro molar and nano molar concentrations of the drug increase the cleavage of Raptor [44]. It has been demonstrated that inhibition of mTOR occurs under different circumstances. For instance, low doses of rapamycin can inhibit growth of cancer cells by repressing mTORC1, and the high doses can affect mTORC2. While nanomolar dosage inhibits mTORC1, inhibition of mTORC2 needs micromolar doses of the drug [45].

In this study, certain concentrations of rapamycin and GSK-2126458, showed an effect in controlling *L*. *major* infection in an *in vivo* model. By testing various concentrations of the drug *in vitro*, we showed that low concentration of the drug (below 400 ng/mL) is not effective to control the promastigote and amastigote viability. Furthermore, rapamycin was able to reduce the growth of *L*. *major* promastigotes by 50%at 10 μg/mL for promastigote and 12 μg/mL for amastigotes. In order to confirm the adverse effects on the parasite as judged by MTT assays, we treated live promastigotes of *Leishmania* parasite with low, intermediate and high doses of rapamycin. The indicated doses were selected based on the IC_50_ concentration of the drug. The ultrastructural examination of the parasites by SEM and TEM electron microscopy revealed a direct parasiticidal effect. High concentrations of the drug inflicted serious harm upon both amastigotes and promastigotes. Also, using rapamycin in microgram doses resulted in size reduction and body destruction of the promastigotes. Formation of intracellular vacuoles within the cytoplasm, as observed by TEM, indicates the undergoing stress. Moreover, the effect of the rapamycin on intracellular parasites revealed the reduction in number of the amastigotes after 24 and 48 h. Other studies also used similar approaches for infected macrophages and promastigotes with scanning SEM and TEM in order to show the changes in cellular shape and size [32,46,47]. To assess the immuno regulatory potentials of rapamycin *in vivo*, we used two therapeutic doses based on the work of Araki *et al*., on mouse CD8^+^ T cell responses to LCMV [35]. We also used the same concentration and treatment regimen for the other two mTOR inhibitors. Our data showed that the higher dose of rapamycin that we employed (10.2 μg/dose/day) dramatically reduces footpad swelling and lesions formation as compared to the control group. Similarly, the high dose of GSK-2126458 ameliorated footpad swelling in comparison with the vehicle group. Further, the accurate number of parasites that had propagated in infected lymph nodes were evaluated and compared by real time PCR [48–50]. We found that the prevented progression of the disease by rapamycin and GSK-2126458 was accompanied by a lowered parasite load in the lymph nodes of the mice that were treated with thee agents.

Remarkably, blood concentrations of rapamycin in mice that were effectively treated with this agent were far less than the doses that directly damaged the parasite *in vitro*. Therefore, we theorized that the observed efficacy of mTOR inhibitors, at indicated doses was owed to their immunomodulatory properties. To address this possibility, we quantified IFN-γ and IL-4 levels in splenocyte cultures in search of a potential Th1 bias [51,52]. As hypothesized, a higher IFN-γ/IL-4 ratio was found, suggesting that treatment with mTOR inhibitors “primes” the immune cells for a protective Th1-skewed response in rapamycin and GSK-2126458 treatment. It is worth mentioning that cytokine levels in splenocyte cultures from KU-0063794-treatd mice was also consistent with immunomodulatory changes; yet, the parasite burden was not significantly lower than that in the vehicle cohort. Therefore, blocking both mTORC1 and mTORC2 by KU-0063794 may not be strong enough to induce a meaningful response in this pre-clinical model.

As stated previously, recent studies have demonstrated that certain doses of rapamycin may augment, rather than suppress, T cell responses [18,35]. Therefore, we investigated the activation status of CD4^+^ and CD8^+^ T cells, key players in cellular immunity to *Leishmania* parasites. Previous studies on nude and athymic BALB/c mice showed the necessity of CD4^+^ and CD8^+^ T cells against leishmanial infection [53]. To further assess the systemic effect of mTOR inhibitor therapy infected lymph nodes and spleens of all mice as well as “uninfected” lymph node (the healthy footpad) were harvested and evaluated cytofluorimetrically. These experiments were relatively limited in scope inevitably due to the number of cells that could be obtained from lymph nodes, especially those draining healthy footpads. Nevertheless, we found that treatment with high-dose mTOR inhibitors may increase CD69 expression by both CD4^+^ and CD8^+^ T cells especially in spleen and non-infected lymph nodes. This is consistent with the finding of Novais *et al*., that infection with *Leishmania* parasites leads to CD8^+^ T cells proliferation [54]. It is not clear at this point how “rapamycin-primed” CD4^+^ and/or CD8^+^ T cells may participate in anti-*Leishmania* immunity in our model infection. Future cell depletion strategies will address this question.

To summarize, we propose that the adjuvant effects of rapamycin and one of the mTOR inhibitor GSK-2126458 can be exploited for the treatment of cutaneous leishmaniasis. Our work has yielded impressive results in a therapeutic setting. It remains to be elucidated whether mTOR inhibitors can be used in rational preventative vaccine design. Future studies will also need to test the effect of rapalogs and dual mTOR/PI3K inhibitors in the context of infection with other strains of *Leishmania* and in other forms of leishmaniasis. Finally, caution needs to be exercised until future comprehensive studies use a wider panel and concentrations of mTOR inhibitors that are already available or will become available. The mode/pathway of cell death, including autophagy, necrosis or apoptosis, will be another direction in future investigations.

## Supporting information

S1 Fig Footpad thickness in mice receiving low-dose mTOR inhibitors. BALB/c mice were infected in footpad with *L*. *major* as in Fig 1A. Twenty-one days later, mice were treated daily with vehicle (n = 9), rapamycin (1.5 μg/dose, n = 9), GSK-2126458 (1.5 μg/dose, n = 9) or KU-0063794 (1.5 μg/dose, n = 9) via the *i*.*p*. route. Footpad swelling measurements before (black color) and after treatment are shown for tested and control groups (blue and red color, respectively) (**A**). Photos were taken of footpads from one representative experiment (**B**). Data are pooled from 3 experiments, each of which typically consisted of at least 3 mice/group. Error bars denote SEM.
(TIF)

S2 Fig No differences in *Leishmania* copy number between vehicle-treated and low dose mTOR inhibitor-treated BALB/c mice. Parasite burden was determined in the popliteal lymph nodes (pLN) of the infected footpad by PCR after 10 days of treatment with vehicle or daily low doses (1.5 μg/dose) of mTOR inhibitors (n = 9). Absolute copy numbers of *Leishmania* RV1-RV2 were evaluated using a standard curve and are presented as number of parasites per ng of gDNA from pLNs. The error bars represent the standard error of the mean (SEM). Statistical analysis was conducted using one-way ANOVA.
(TIF)

S3 Fig The effect of treatment with low-dose mTOR inhibitors on the capacity of splenocytes to secrete cytokines. Splenocytes that were prepared at the end of the experiment were stimulated with *Leishmania* lysate or anti-CD3 mAb, and culture supernatants were collected on days 1 and 3, in which IL-4 (**A**) and IFN-γ (**B**) levels were quantified. The IFN-γ/IL-4 ratio was also evaluated (**C**). No significant changes were detected. Error bars represent standard error of the mean (SEM). Statistical differences were calculated, using a Student’s *t*-test. n = 9 (pool of three different experiments).
(TIF)
