# Supplementary figures and images for: Therapeutic control of leishmaniasis by inhibitors of the mammalian target of rapamycin
Source: PLoS Negl Trop Dis. 2018 Aug 22;12(8):e0006701. doi: 10.1371/journal.pntd.0006701 (PMC6122837; doi:10.1371/journal.pntd.0006701)

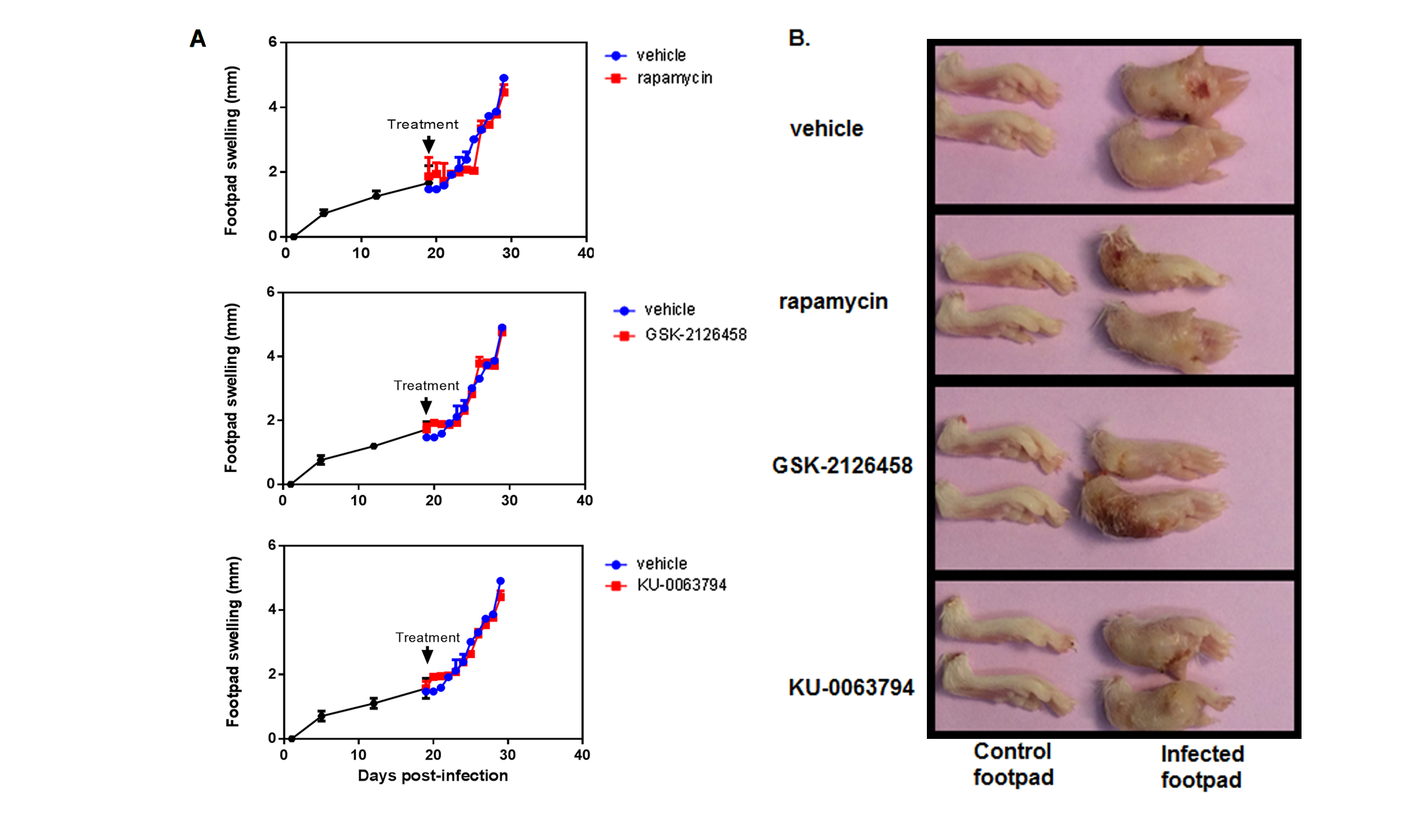

Supplement: S1 Fig — BALB/c mice were infected in footpad with L. major as in Fig 1A. Twenty-one days later, mice were treated daily with vehicle (n = 9), rapamycin (1.5 μg/dose, n = 9), GSK-2126458 (1.5 μg/dose, n = 9) or KU-0063794 (1.5 μg/dose, n = 9) via the i.p. route. Footpad swelling measurements before (black color) and after treatment are shown for tested and control groups (blue and red color, respectively) (A). Photos were taken of footpads from one representative experiment (B). Data are pooled from 3 experiments, each of which typically consisted of at least 3 mice/group. Error bars denote SEM. (TIF) [file pntd.0006701.s001.tif]

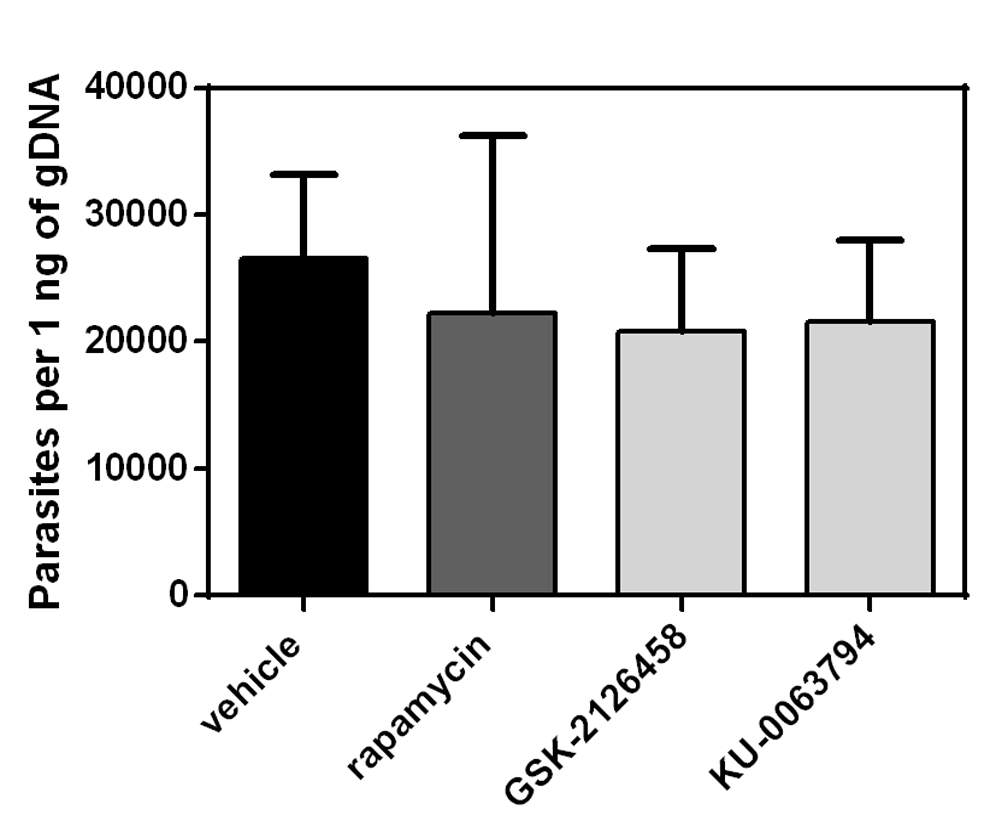

Supplement: S2 Fig — Parasite burden was determined in the popliteal lymph nodes (pLN) of the infected footpad by PCR after 10 days of treatment with vehicle or daily low doses (1.5 μg/dose) of mTOR inhibitors (n = 9). Absolute copy numbers of Leishmania RV1-RV2 were evaluated using a standard curve and are presented as number of parasites per ng of gDNA from pLNs. The error bars represent the standard error of the mean (SEM). Statistical analysis was conducted using one-way ANOVA. (TIF) [file pntd.0006701.s002.tif]

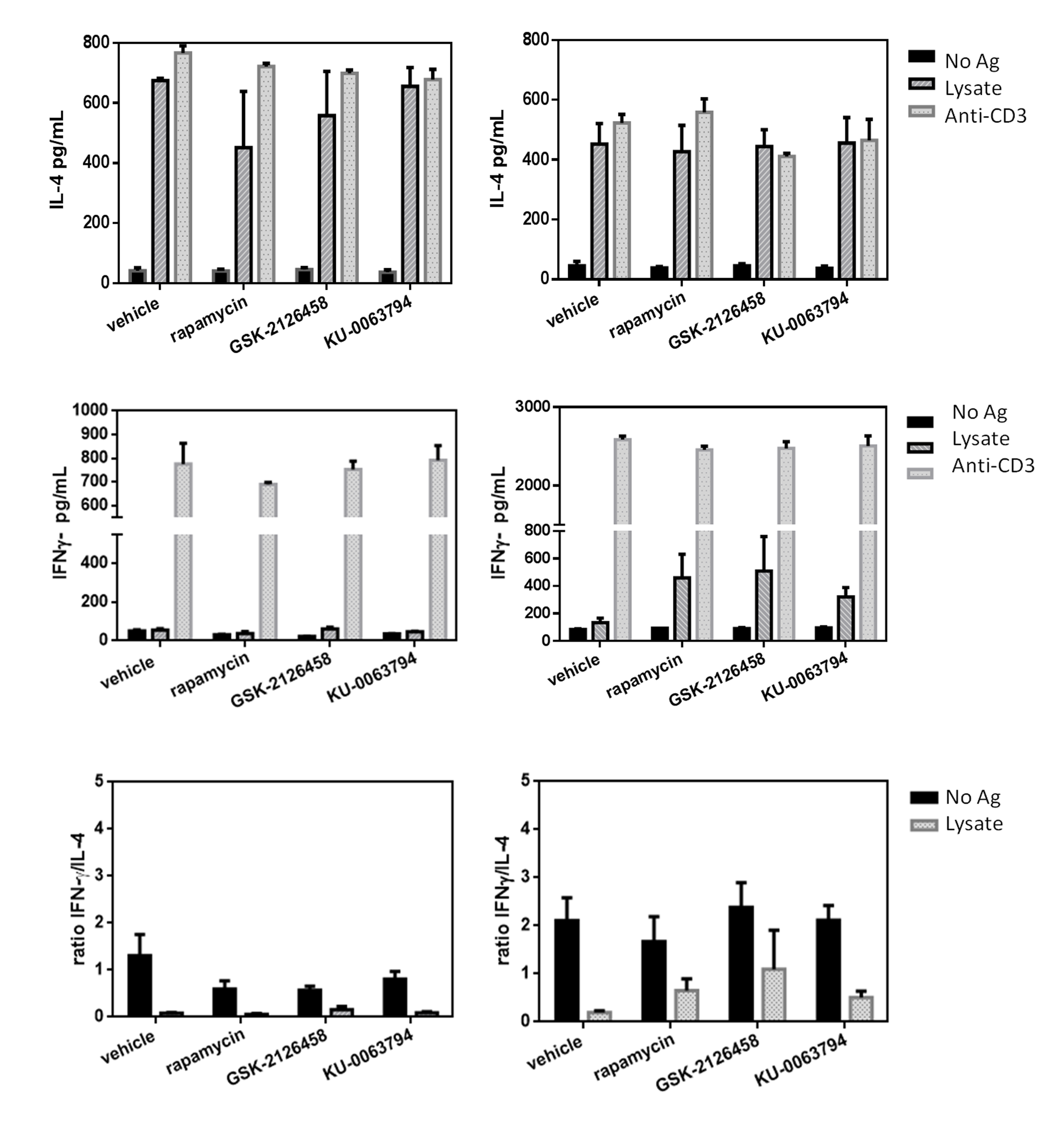

Supplement: S3 Fig — Splenocytes that were prepared at the end of the experiment were stimulated with Leishmania lysate or anti-CD3 mAb, and culture supernatants were collected on days 1 and 3, in which IL-4 (A) and IFN-γ (B) levels were quantified. The IFN-γ/IL-4 ratio was also evaluated (C). No significant changes were detected. Error bars represent standard error of the mean (SEM). Statistical differences were calculated, using a Student’s t-test. n = 9 (pool of three different experiments). (TIF) [file pntd.0006701.s003.tif]
